# Calorimetric Studies of Alkali-Activated Blast-Furnace Slag Cements at Early Hydration Processes in the Temperature Range of 20–80 °C

**DOI:** 10.3390/ma14195872

**Published:** 2021-10-07

**Authors:** Aleksandr Usherov-Marshak, Danutė Vaičiukynienė, Pavel Krivenko, Girts Bumanis

**Affiliations:** 1 Faculty of Civil Engineering, Kharkiv National University of Civil Engineering and Architecture, Sumska Str. 40, 61002 Kharkiv, Ukraine; a.v.usherov@gmail.com; 2 Faculty of Civil Engineering and Architecture, Kaunas University of Technology, Studentu St. 48, LT-51367 Kaunas, Lithuania; girts.bumanis@rtu.lv; 3 Scientific Research Institute for Binders and Materials, Kyiv National University of Construction and Architecture, Povitroflotskyi Pr. 31, 03037 Kyiv, Ukraine; pavlo.kryvenko@gmail.com

**Keywords:** alkali-activated cement, blast-furnace slag, crystallo-chemical hardening accelerator, heat release, heat curing

## Abstract

In the hydration process of inorganic cements, the analysis of calorimetric measurements is one of the possible ways to better understand hydration processes and to keep these processes under control. This study contains data from the study of thermokinetic processes in alkali-activated blast-furnace slag cements compared to ordinary Portland cement (OPC). The obtained results show that, in contrast to OPC, the heat release values cannot be considered as a characteristic of the activity of alkali-activated blast-furnace slag cements. In addition, it is concluded that in the case of OPC cements, cumulative heat release is a criterion for the selection of effective curing parameters, while in the case of alkali-activated blast-furnace slag cements, a higher heat rate (which increases sharply with increasing temperature from 20 to 40 °C) is a criterion. From the point of views of thermokinetics, the rate of heat release at temperatures up to 40 °C can be a qualitative criterion that allows to choose the parameters of heat curing of alkali-activated cement concretes. By introducing a crystallo-chemical hardening accelerator, such as Portland cement clinker, into the composition of alkali-activated blast-furnace slag cements, it is possible to accelerate the processes not only in the condensation-crystallization structure formation stage, but also in the dispersion-coagulation structure formation stage. Portland cement clinker increased the efficiency of thermal curing at relatively non-high temperatures.

## 1. Introduction

In recent years, alkali-activated cements have become a viable ecological alternative to traditional cementitious materials. They also have good durability, mechanical strength, thermal properties and reduced carbon footprint. Compared with ordinary Portland cement (OPC), alkali-activated cements are more environmentally friendly, and the raw materials are usually industrial waste, which can contribute to the waste disposal. The common raw materials of these binders could be a wide range of the industrial aluminosilicate byproducts or wastes, such as slag, fly ash, bottom ash, zeolitic waste, glass powder [1,2].

Despite a great number of works dedicated to colorimetric studies [3,4,5,6,7,8], some hydration aspects of alkali-activated cements are not clear. The initial interaction stages of the aluminosilicate component with the highly alkaline (pH > 11.5) liquid phase at normal and elevated temperatures are important. At these stages, the interrelationships between flowing processes and many factors related to cement composition and temperature could be most revealed. To better understand the nature of hydration processes, it is necessary to analyze the results of calorimetric measurements: the amount of heat released and the rate of heat evolution.

Calorimetry of OPC cement is quite well studied [9,10,11,12]. However, as shown by calorimetric studies of alkali-activated cements, its interpretation cannot be automatically applied to these cements due to hydration processes. This is due to the different mechanisms of structure-forming processes in the OPC cements and alkali-activated cements. Therefore, the solid OPC cement phase, which consists of a mixture of many basic minerals with a high degree of ionic bonding (Ca-O-Si), is destroyed by protonating them in the presence of mixing water [13]. The solid phase of the alkali-activated cement is reflected by low base phases with a high degree of Si-O-Si and Al-O-Al covalent bonds. When water was incorporated, ionic forces are not strong enough to decompose the solid phase. Therefore, the destruction of covalent bonds was only possible under very alkaline conditions. (pH > 11.5) [14].

In contrast to OPC-based cements, alkali-activated cements have an inverse relationship between strength and heat release [15,16]. The hydration of alkali-activated cements is accompanied by low heat of hydration, but at an early age their strength is quite high [17,18]. Several factors affect their interrelation, and the amount of alkaline activator is one of these factors [19]. The type of alkaline activator is important as well [20,21].

It is known that with increasing water content in cement paste, the hydration of Portland cement increased as well [22]. However, as could be seen from [5], the increase of the water-to-cement or slag ratios from 0.45 to 0.6 in the alkali-activated slag cements has no significant effect on the induction period and the general trend of heat evolution of the OPC, but it makes the accelerated hydration peak slightly lower. The ratio of water to solids does not affect the rate of heat evolution.

Temperature has a significant effect on cement hydration processes. It can be assumed that due to the different hydration mechanism of OPC and alkali-activated cements, this effect may be different. This assumption is supported by several researchers [5,6,23,24,25,26]. However, the lack of data on the interaction between the thermokinetic characteristics of alkali-activated cement and the development of early strength do not allow to predict at least the optimal curing parameters of alkali-activated cement concretes at elevated temperatures.

In the process of cement hydration, the addition of crystallo-chemical hardening accelerators is important as well for the acceleration of condensation-crystallization structure formation [4,14,27]. However, there are no data on their influence on the initial stages of dispersion-coagulation structure formation, as well as on the thermokinetic characteristics of alkali-activated cements.

The aim of the study was to perform calorimetric studies of hydration processes at the early stages of alkali-activated blast-furnace slag cements in the temperature range of 20–80 °C compared to OPCs, and to substantiate the appropriate criteria for selecting optimal thermal curing parameters when using alkali-activated blast-furnace slag cement concrete. A blast-furnace slag was chosen for the study as the most widely used aluminosilicate component of the alkali-activated cements.

## 2. Materials and Methods

### 2.1. Raw Materials

A ground granulated blast-furnace slag (further-slag) was used as the major aluminosilicate component and calcium source as well (Table 1). Slag contains calcium oxide 44.0%, silica 37.2%, alumina 8.8% and magnesia 5.5%. Portland cement clinker in a quantity of 10% by mass (further-clinker) was used as a crystallo-chemical hardening accelerator. Its chemical composition is shown in Table 1.

**Figure 1:**
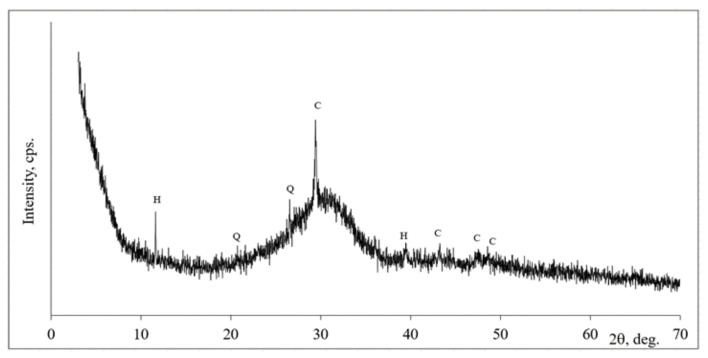
The X-ray diffraction patterns of slag.

According to mineral composition of slag the crystalline minerals, such as hydrotalcite, quartz, and calcite dominated (Figure 1). In addition to the aforementioned crystalline compound’s amorphous part related with a broad hallow on XRD graphic within 2θ degree range in 20–35° was detected as well.

Notes: H is hydrotalcite-Mg_6_Al_2_CO_3_(OH)_16_·4H_2_O (14-191), Q is quartz SiO_2_ (78-2315), C is calcite CaCO_3_ (72-1651).

The dry mixes of cement components were ground to a specific surface area of 4000 cm^2^/g by Blaine. After that the cement components (containing 90% or 100% slag by mass) were mixed with water or the solution of Na_2_O·SiO_2_·5H_2_O with a density of 1250 kg/m^3^. The ratio of solid and liquid components was 0.5.

Properties of the alkali-activated blast furnace slag cements were in compliance with the standard of Ukraine DSTU B V.2.7-181:2009, Cement LCEM I, Class 800.

### 2.2. Examination and Testing Techniques

Thermokinetic measurements were performed using a differential Tian-Calvet-type calorimeter [28]. The procedure of the experiment was as described in [29]. In all experiments, the cement sample was 1 g. The calorimetric experiment covered three periods: initial, main, and final.

In the initial period, the calorimetric system temperature is equalized before the heat of hydration measurements. The ingredients (the cements and liquid) were mixed at room temperature and after that they were heated to desired temperatures to provide heat release. The final period begins when the measured heat ends. The heat of hydration (H1) is calculated as the difference between the heat release of unhydrated (Qa) and hydrated (Q1) cement samples.

Two kind of experiments has been performed: hydration of cement paste and dissolution of binders and hardened paste in acid. The test samples were dissolved in a mixture of acids. The liquids used for the calorimetric measurements were the mixture of nitric and fluoric acids obtained by mixing the following acids: 2M · HNO_3_ and HF (concentration = 50%) to obtain the following final concentrations: 1.95 M of HNO_3_ and 0.5 M of HF. First of all, the heat release of unhydrated cement samples were performed during experiments measuring. The sample of 10 mg cement was dissolved in 1400 mg of calorimetric liquid (the above described mixture of nitric and fluoric acids) and the rate of heat release during the hydration process and the cumulative heat release were measured.

The experiments were performed after 7 days of curing in a hermetically sealed container at normal temperature (20 ± 2 °C). The hardened cement paste was ground to produce a powder and a sample of 14 mg was taken. In order to take measurements of the heat release, the quantity of the hydrated cement paste samples was increased by 40% compared to that of the non-hydrated cement sample. A quantity of the unhydrated cement was increased in order to obtain their equal quantities calculated on a solid phase with account of its hydration (0.4). In further calculations, the measured values of the heat release were re-calculated for 1 g of the unhydrated cement. The conditions of the experiment were as follows: duration of the experiment—30 min, temperature—25 °C. The formation processes of cements structure were studied using a resonance device described in [28].

To reveal effect of curing on thermokinetic characteristics, the influence of the following parameters was studied: time of temperature application, rate of heating, duration of isothermal heating, and temperature of heating.

## 3. Results and Discussions

### 3.1. The Thermokinetics of Cement Hydration Process

**Figure 2:**
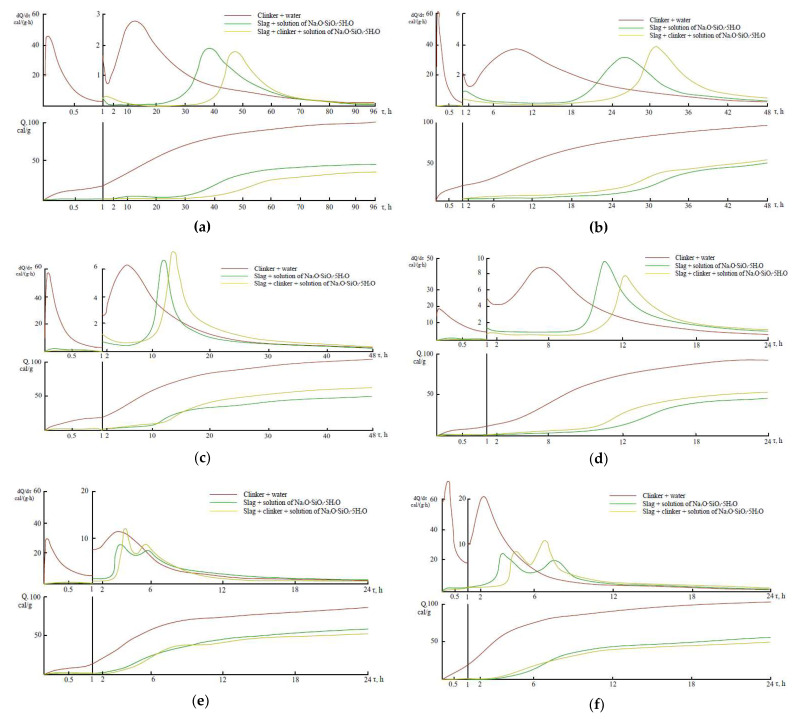

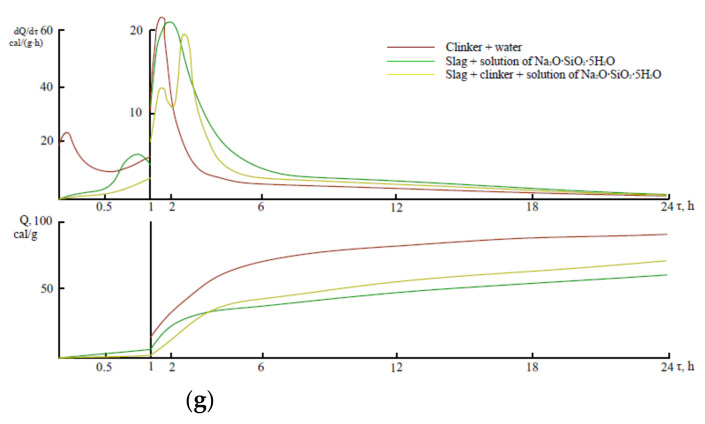
The thermokinetic characteristics of cements hydration process at temperatures of 20 °C (**a**); 30 °C (**b**); 40 °C (**c**); 50 °C (**d**); 60 °C (**e**); 70 °C(**f**); 80 °C (**g**).

The calorimetric studies result of cements hardened at various temperatures are given in Figure 2 and Table 2, Table 3, Table 4. In this study three systems were investigated.

#### 3.1.1. The System of Portland Cement Clinker and Water

In hydration products high basic compounds are formed due to the high initial activity of OPC clinker. In this case, Ca(OH)_2_, calcium silicate and aluminate hydrates formed. The formation of these compounds is accompanied by high heat release [29]. In addition, the products of the basic hydration recrystallize to low basic, which is also accompanied by heat release due to the reduction in energy during the transformation to a more stable state.

The first exothermic peak, formed by wetting of finely dispersed clinker powder in the liquid phase (with water), had high intensity and induction period is short (after 5.2 h at t = 20 °C; after 1.9 h at t = 40 °C; after 1.5 h at t = 60 °C, and after 0.9 h at t = 80 °C). The second exothermic peak is characterized by the early onset (after 13 h at t = 20 °C; after 64 h at t = 40 °C, after 3.34 h at t = 60 °C, and after 2 h at t = 80 °C) and it had high-rate values of heat release (2.8 cal/(g·h) at t = 20 °C and 9.1 cal/(g·h) at t = 50 °C). It significantly increased values of heat release: 99 cal/g after 96 h at t = 20 °C; 92 cal/g after 24 h at t = 50 °C. All these is characteristics of the thermokinetic measurements shown in Figure 2. Rapid hydration does not cause high strength values during the initial hydration period, which is attributed to the weak Ca-O-Si type ionic bonds of the high-basic hydration products, coarse crystalline structure, and porosity of the obtained cement stone [30].

#### 3.1.2. The System of Slag and Na_2_O·SiO_2_·5H_2_O Solution

Granulated blast furnace slag is a low-basic aluminosilicate that includes hardly polarized covalent bonds, and its interaction with water is much weaker compared to OPC [13]. For this reason, the thermodynamic activity of the solid slag phase when mixed with water is very low. However, the structure formation mechanism of alkali-activated cements, in contrast to OPC, is explained not by protonation of ionic bonds but by covalent Si-O-Si and Al-O break. In this study the alkali metal compound, Na_2_OSiO_2_·5H_2_O, provides for the “loosening” of the slag structure in the initial interaction phases, but in the later stages, the formation of alkaline aluminosilicate hydrates with few basic alkalis and calcium silicate hydrates, which are the main compounds of hydration products [4]. Several basic compounds are formed with negligible heat release. The distinctive feature of these cements hydration is that there is no first exothermic peak (approximately 1–1.3 cal/g), a long induction period (35 h at t = 20 °C; 20 h at 30 °C; 11 h, when t = 40 °C; 9 h, when t = 50 °C; 3 h, when t = 60 and 70 °C; 2.25 h, when t = 80 °C) with low and almost constant heat release rate, relatively short acceleration period (28 h, when t = 20 °C; 11 h, when t = 30 °C; 5 h, when t = 40 °C, 4 h, when t = 50 °C, 3 h, when t = 60 °C, 5 h, When t = 70 °C, 0.5 h = 80 °C), during which the main hydration reactions are very intense.

At a temperature of 60–70 °C, the so-called “splitting” of the second exothermic effect can be observed only in purely alkali activated cement, whereas at t = 80 °C only in the alkali activated cement with additive of clinker. A value of the second exothermic effect of two alkali activated cements in principle (fundamentally) does not differ from that of the Portland cement. A longer induction period results in cumulative heat release at the beginning of the process, but a further increase in heat release rate over an accelerated period results in higher cumulative heat release values.

The comparative analysis results of the thermokinetic hydration processes of OPC and alkali-activated cement led to the conclusion that the induction period of alkali-activated cement was longer (after 30 h at 20 °C; after 17 h at 30 °C; after 10 h at 40 °C; after 6 h at 50 °C; after 1.5 h at 60 and 70 °C; after 1.25 h at 80 °C) and the lower values of cumulative heat release during 24 h (60 cal/g at 20 °C; 41 cal/g at 40 °C; 33 cal/g at 60 °C; 22 cal/g at 80 °C). Despite the revealed regularities, the value of the second exothermic effect in the case of alkali-activated cement is quite high, and its onset time at high temperature does not differ from that of OPC.

As written previously, the difference in thermokinetic characteristics of alkali-activated cement and OPC cement can be attributed to a fundamentally different mechanism of hydration. It is obvious that the formation of low alkali compounds in the hydration process of alkali-activated cements takes place in later stages and with lower heat release. In contrast, in OPC cements, the formation of high-basic calcium aluminate hydrates and calcium silicate hydrates occurs in the early stages of hydration, which is accompanied by considerable heat release.

#### 3.1.3. The Systems of Slag, Portland Cement Clinker and Solution of Na_2_O·SiO_2_·5H_2_O

The addition of clinker as a hardening accelerator promotes an increase in the rate of crystal structure formation in the condensation-crystallization structure formation stage. Much earlier beginning of the appearance of the second exothermic peak (10 h at t =20 °C; 2 h at t =40 °C; 0.5 h at t =60 °C; 0.7 h at 80 °C) observed in thermokinetic curves, but the values of the second exothermic peak and the cumulative heat release of the compositions with clinker are similar to the values of cement compositions without clinker additive, but the strength of the samples is significantly increased due to the intensification of hardening processes by adding the hardening accelerator (clinker) [29].

**Figure 3:**
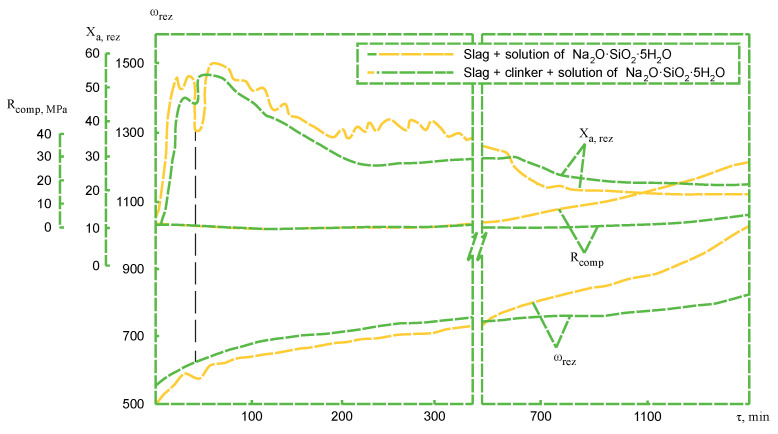
The influence of the additive (Portland cement clinker) on the structure formation process at temperature of 20 °C.

This is confirmed by the study results of hardened cement paste structure formation obtained using a resonant device [30]. In the study the amplitude-frequency characteristics (range 1–2) tend to increase immediately after the slag is mixed with Na_2_O·SiO_2_·5H_2_O solution (Figure 3). The increase of the resonance frequency (ω) values confirms the formation of the colloid structure in the alkali-activated slag cement dispersion. The increase in the resonance amplitude (X_a,res_) occurs due to the surface layers destruction of slag in the process of interaction slag with the alkaline medium. The addition of clinker leads not only to the intensification of the formation processes of the dispersion-coagulation structure (more intensive growth of values (X_a,res_) in the initial hydration stage), but also to the accelerated formation of the precursors of the crystalline phase. This is confirmed by the increase in frequency resonance (ω_res_) values and the intensive increase in cement stone compressive strength values (R_comp_).

### 3.2. Influence of Temperature on Heat Release during Accelerated Curing

Each time the temperature rises by 10 °C, the rate of homogeneous chemical reaction is 2–4 times increased. The interrelations that reflect the effect of temperature on the reaction rate become simpler, given the dependence of the logarithm of the rate constant on the inverse of the absolute temperature value. The logarithm of the reaction rate constant ln(k) depends almost linearly on the value (1/t). The required dependence, under certain assumptions, could be expressed by the Arrhenius equation (1):

ln(k) = A/t + B(1)

where k—reaction rate constant; A and B—individual constants for a given reaction; t—temperature.

**Figure 4:**
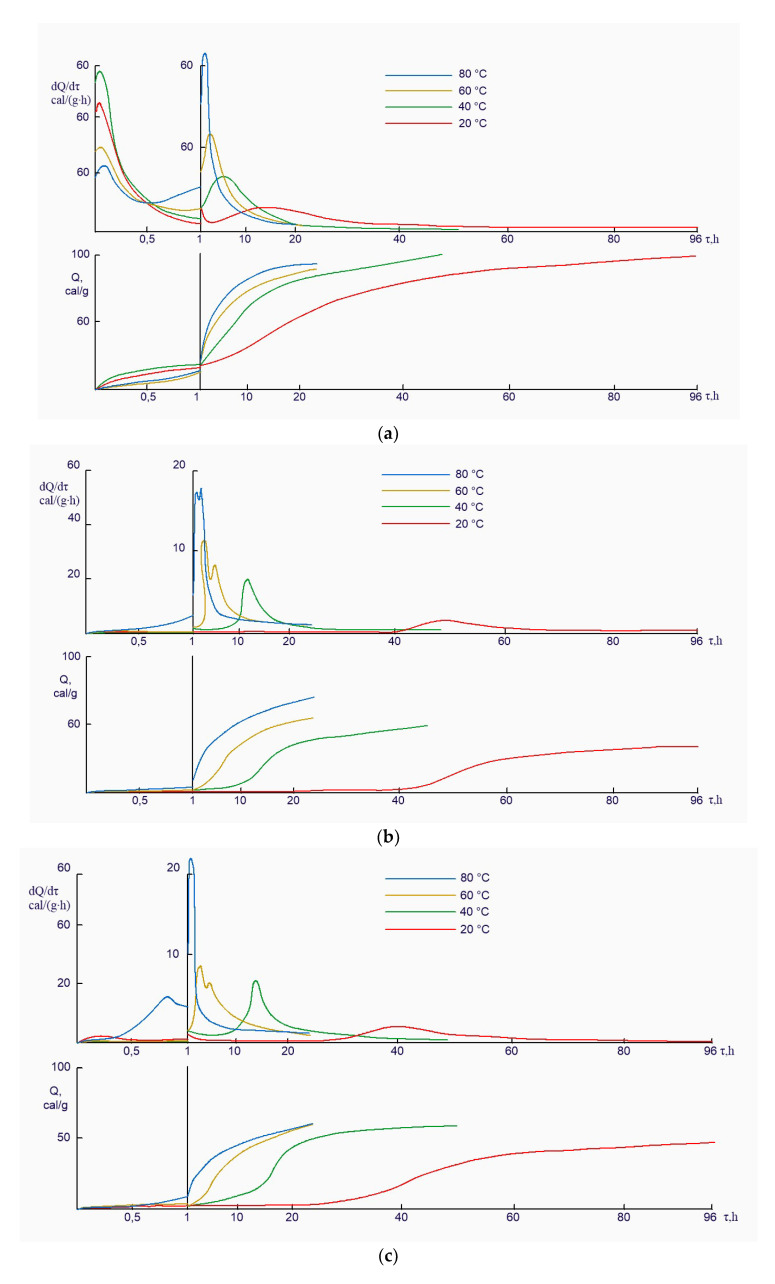
The comparative thermokinetic characteristics of cement hydration process at curing temperatures and in the systems: clinker + water (**a**); slag + solution of Na_2_O·SiO_2_·5H_2_O (**b**) and slag + clinker + solution of Na_2_O·SiO_2_·5H_2_O (**c**).

This assumption is confirmed by the data of calorimetric studies (Figure 4). As the temperature increased from 20 to 80 °C, all the tested samples showed an intensification of the hydration reactions and, respectively, an increase in the value of heat release and cumulative heat release. This is particularly clear in the example of alkali-activated slag cement, the hydration of which tended to accelerate abruptly with a rise in temperature from 20 to 40 °C. At the same time, it is shown that the increase in temperature from 60 to 80 °C does not strongly affect these characteristics.

### 3.3. Influence of Curing on Thermokinetic Characteristics

Choice of optimal curing regimes of concrete is restricted, usually, by mixture composition, sustainability, strength, minimal duration of curing before temperature application, maximal allowed heating rates, duration of isothermal heating and other factors.

**Figure 5:**
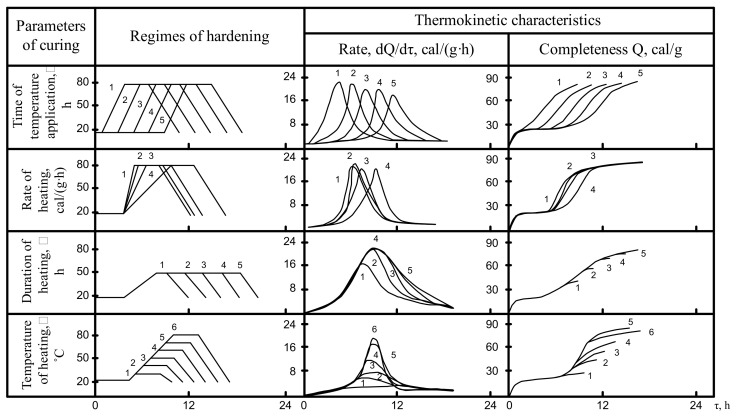
The influence of heat curing parameters on thermokinetic characteristics of the cement hydration in the clinker + water system.

**Figure 6:**
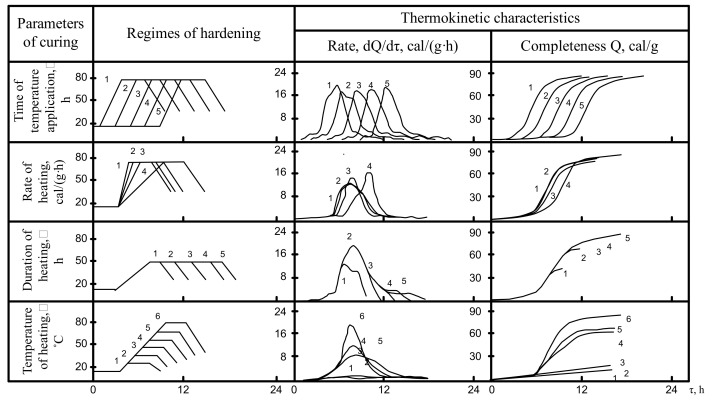
The influence of heat curing parameters on thermokinetic characteristics of the cement hydration in the slag + solution of Na_2_O·SiO_2_·5H_2_O system.

**Figure 7:**
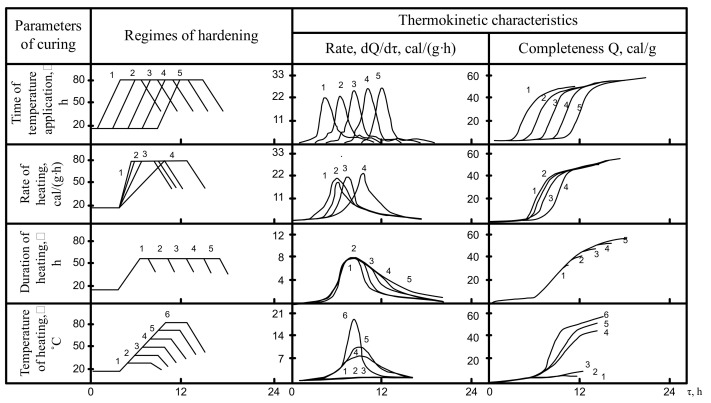
The influence of heat curing parameters on thermokinetic characteristics of the cement hydration in the slag + clinker + solution of Na_2_O·SiO_2_·5H_2_O system.

The data analysis on the changes in the thermokinetic characteristics of the tested cement under certain non-isothermal conditions (Figure 5, Figure 6, Figure 7) shows that with the longer curing duration of hardening cement mixes varying from 1 to 5 h before application of temperature the lowering of the rate of heat release is observed only for the OPC-based cements. In the case of the alkali-activated cement “slag + solution of Na_2_O·SiO_2_·5H_2_O” the duration of curing before application of temperature does not affect the rate of heat release. At the same time, for the cement composition “slag + clinker + solution of Na_2_O·SiO_2_·5H_2_O” with the longer duration of curing before the application of temperature the rate of heat release increases.

The time during which a maximum rate of heat release is achieved for all cements under study is shifted to the right along the axis of time proportionally to the moment of application of temperature. This can be attributed to the specific features and elementary constituents of the hydration process: adsorption, dissolution, formation of precursors of crystals, crystallization, etc. Especially important is that some reduction of the complexity of heat release in the beginning of hydration is changed, judging from the dependence Q = f(T), by approximating the values of the complexity of the heat release to each other as soon as after 10–12 h.

When the heating rate reaches 10–24 cal/(g·h), the maximum heat release also shifts to the right along the time axis. However, hydration of alkali-activated cements shows an increase in rate and complexity as the heating rate slows. At a heating rate of 10 cal/(g·h)**,** the value of the exothermic peak is minimal for OPC cement. The hydration of these cement compositions “slag + solution of Na_2_O·SiO_2_·5H_2_O· and “slag” + clinker + solution of Na_2_O·SiO_2_·5H_2_O” reaches its maximum.

The influence of heating time on isothermal heating at 80 °C is difficult to understand. At this high temperature the maximum heat release is achieved before the heating is completed, and further curing promotes only an insignificant increase in the cumulative heat release. When the heating temperature is 20–30 °C lower the influence of the curing time is even clearer: in case of a short curing time (2 h) the sharp lowering rate of heat release is observed. For a longer curing period of up to 6 h the process is more complete and a further increase in duration promotes only an insignificant increase in heat release.

In the case of non-isothermal thermokinetics, the limit value of the heating temperature plays an important role. The value of the absolute value of the maximum heat release increases in proportion to the curing temperature. The time taken to reach it for the cements under study is similar. The change in the complexity of hydration depends on the applicable isothermal conditions.

The analysis of the obtained data allowed to conclude about the essential influence of heat curing parameters on the thermokinetic characteristics of hydration rate and complexity of the studied cements.

### 3.4. The Substantiation Criterion for the Choice of Heat Curing parameters

A complex set of thermokinetic studies performed, supported by collected data on the synthesis of hardened cement strength and the results of physico-chemical studies of hydration products, can provide a theoretical basis for determining the criterion of heat efficiency of concrete curing.

As can be seen from the performed experiments, the higher basicity of the cement, the higher the amount of low basic calcium silicate hydrates in the hydration products. This is accompanied by a decrease in total heat release during the hydration process of the cement and an increase in the strength of the hardened cement.

**Figure 8:**
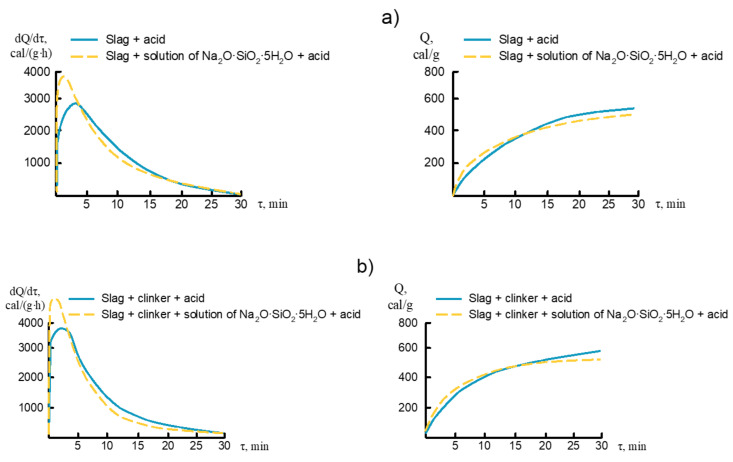
Interrelationships dQ/dτ−f(τ) and Q−f(τ) in the process of dissolution of the unhydrated (**a**) and hydrated cement samples (**b**). The “cement + acid” is unhydrated and “cement + water glass + acid” is hydrated.

The results of calorimetric studies were based on studies of thermochemical methods. The heat release of both unhydrated and hydrated cement samples was measured by the dissolution calorimetry method (Figure 8).

In the light of thermokinetics, the heat release of a substance is identical to the heat required for its formation. Interesting is the comparison of hydration heat calculated from thermochemical experiments and the heat of hydration measured directly by thermokinetic studies.

In general, the complex analysis of heat release in the hydration process of the investigated cements provides information on the reaction possibilities of the cement and the mechanism of hydration. The 1-day heat values of accumulated heat and 7-days of hydration are quite high. Heat release for 7-days is significantly higher than for 1-day, indicating a long duration of the hydration reaction. Alkali activated cement is characterized by low and almost similar heat release values obtained also by thermochemical and thermokinetic experiments. The results are also interesting in assessing and considering the heat released in the process of cement hydration in the production of concretes from them [31,32].

Thus, it can be concluded that the efficiency of high basic cements in the process of curing is determined by the amount of heat released during the hydration process, which plays an important role in preheating the concrete mix and can contribute to the heat balance of the curing process.

The mechanism of hydration of so-called low basic cements (the cements with the lower basicity of a solid phase compared to that of traditional cements; an example is the alkali-activated cement) [16]. Its hydration is completely different and is caused not by the protonization of ionic bonds, but by the break of covalent Si-O-Si and Al-O-Al bonds, followed by the synthesis of low hydration phases from the destruction products resulting in activation of the hydration process when the temperature rises from 20 to 40 °C.

**Figure 9:**
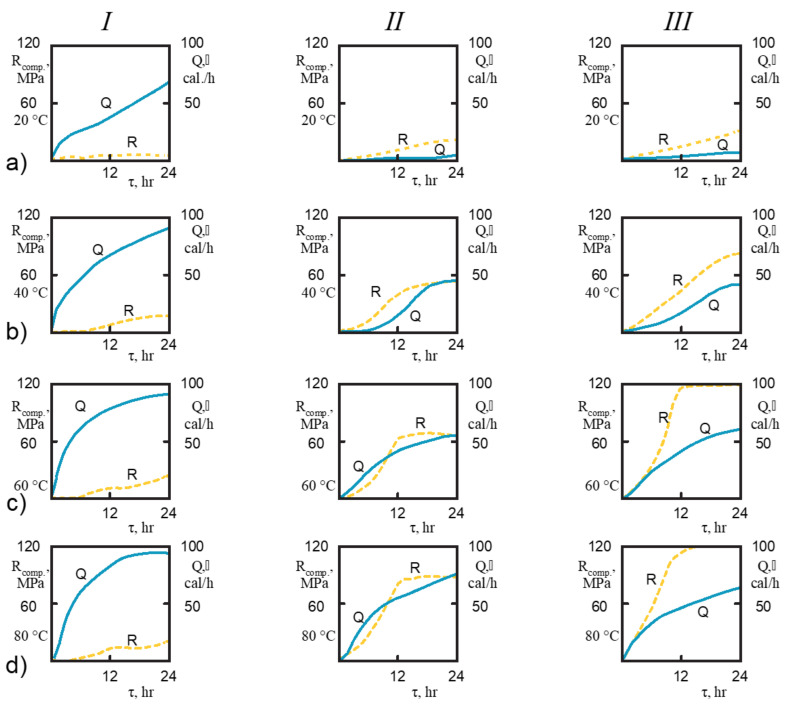
Heat release Q and compressive strength R_comp_ of the cements:(**a**) t = 20 °C; (**b**) t = 40 °C; (**c**) t = 60 °C; (**d**) t = 80 °C.

The strength analysis of hardened cement paste after curing at a relatively low temperature (below 40 °C) and the accumulating·heat release of the cement in the early stages of hydration (Figure 9) showed that the cement hydration process was activated. In this case low basic cement has a higher heat release rate. From a thermokinetic point of view, the rate of heat release at 40 °C can be a criterion for the efficiency of such systems in the thermal curing process.

Notes: I—“clinker + water”; II—“slag + solution of Na_2_O·SiO_2_·5H_2_O”; III—“slag + clinker + solution of Na_2_O·SiO_2_·5H_2_O”.

By adding the clinker in the cement composition, the mechanism of hydration was determined similar to hydration under normal conditions. It intensifies the formation of dispersion-coagulation thixotropic structure based on condensation–crystallization structures [23,27,30,33].

**Figure 10:**
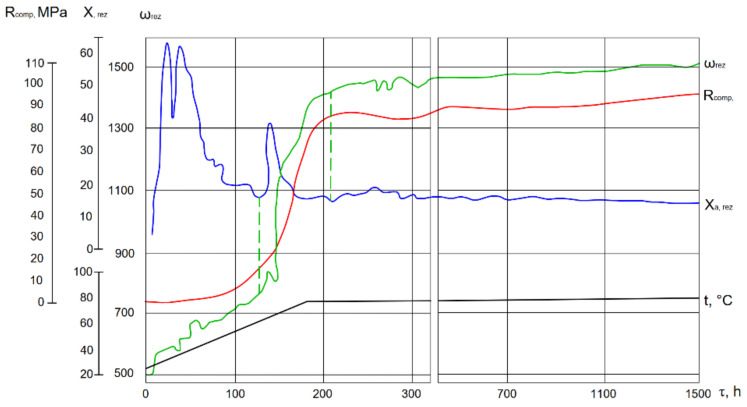
The influence of Portland cement clinker on the structure formation of the cement in the system slag + clinker + solution of Na_2_O·SiO_2_·5H_2_O cured at temperatures from 20 to 80 °C.

This is confirmed by the higher resonant frequency values (ω_res_) (Figure 10), which confirm that the development of hydration processes in this case is much more intense at significantly lower temperatures than required for cements without Portland cement clinker.

## 4. Conclusions

The thermokinetic regularities of the alkali-activated blast-furnace slag cement compared to OPC during·hydration process were studied. It was proved that in the case of alkali-activated blast-furnace slag cement the heat release values are not characteristic of cement activity, as in the case of OPC. Therefore, in the case of OPC the cumulative heat release is a criterion for the selection of effective curing parameters. In the case of alkali activated blast-furnace slag cement the heat release rate, which increases sharply with increasing temperature from 20 °C to 40 °C were determined.

From the viewpoint of thermokinetics, the heat release rate of alkali-activated blast-furnace slag cement at temperature of 40 °C can be a qualitative criterion that allows to choose the parameters of heat curing for alkali-activated blast-furnace slag cement concretes.

It has been found that with the addition of a crystallochemical hardening accelerator in the composition of alkali-activated cement, the processes are accelerated not only by forming a condensation–crystallization structure, but also by forming a dispersion-coagulation structure as well.

## Figures and Tables

**Table 1:** The chemical composition of raw materials.

Materials	Composition, % by Mass						
	SiO_2_	Al_2_O_3_	CaO	MgO	Fe_2_O_3_	SO_3_	NaO + K_2_O
Clinker	23.0	6.5	63.0	2.5	3.0	1.0	1.0
Slag	37.2	8.8	44.0	5.5	1.5	3.0	0.0

**Table 2:** The thermokinetic characteristics for the hydration process of clinker and H_2_O.

Curing Temperature t, °C	Time During Which Maximal Rate of Heat Release τ is Reached, h			Rate of Heat Release dQ/dτ, cal/(g·h)			Cumulative Heat Release Q, cal/g			
	1 max	2 max	3 max	1 max	2 max	3 max	12 h	24 h	48 h	96 h
20	0.11	13.0	-	45	2.8	-	35	63	85	99
30	0.1	9.5	-	62	3.8	-	50	78	99	-
40	0.05	6.0	-	57	6.2	-	68	89	103	-
50	0.06	5.5	-	17	9.1	-	74	92	-	-
60	0.1	3.3	-	30	11.7		77	88	-	-
70	0.2	2.25	-	36	20.9.	-	95	106	-	-
80	0.08	2.0	-	24	21.0	-	84	94	-	-

**Table 3:** The thermokinetic characteristics for the hydration process of the cement slag and Na_2_O·SiO_2_·5H_2_O solution.

Curing Temperature t, °C	Time During Which Maximal Rate of Heat Release τ is Reached, h			Rate of Heat Release dQ/dτ, cal/(g·h)			Cumulative Heat Release Q, cal/g			
	1 max	2 max	3 max	1 max	2 max	3 max	12 h	24 h	48 h	96 h
20	0.16	48.0	-	1	1.7	-	2.7	3	13	34
30	1.5	26.0	-	1	3.4	-	5.8	15	52	-
40	0.75	13.5	-	1.2	7.4	-	11.0	48	65	-
50	0.75	10.7	-	1.3	9.4	-	27	55	-	-
60	-	3.7	5.5	-	11.8	8.6	44	55	-	-
70	-	3.6	7.5	-	8.2	6.8	41	52	-	-
80	-	1.5	2.5	-	13.2	19.8	55	72	-	-

**Table 4:** The thermokinetic characteristics for the hydration process of the cement slag, clinker and Na_2_O·SiO_2_·5H_2_O solution.

Curing Temperature t, °C	Time During Which Maximal Rate of Heat Release τ is Reached, h			Rate of Heat Release dQ/dτ, cal/(g·h)			Cumulative Heat Release Q, cal/g			
	1 max	2 max	3 max	1 max	2 max	3 max	12 h	24 h	48 h	96 h
20	0.25	38.0	-	1.7	1.9	-	2.9	4.5	30	47
30	0.25	31.0	-	0.8	4.1	-	4.9	8.8	50	-
40	0.25	11.6	-	1.4	6.7	-	16.5	38	52	-
50	0.16	12.3	-	0.5	7.7	-	12.7	46	-	-
60	-	3.25	5.5	-	8.6	7.0	46	60	-	-
70	0.75	4.6	6.8	1.2	8.5	11.0	45	59	-	-
80	-	0.8	1.5	-	14.9	22.0	46	61	-	-

## Data Availability

Data Sharing is not applicable.
